# Integrated Psychological Services in Pediatric Oncology: Caregiver Perspectives at Diagnosis

**DOI:** 10.3390/cancers16183137

**Published:** 2024-09-12

**Authors:** Emily Bernstein, Anna M. Jones, Niki Jurbergs, Jennifer L. Harman, Sean Phipps, R. Elyse Heidelberg

**Affiliations:** Department of Psychology and Biobehavioral Sciences, St. Jude Children’s Research Hospital, Memphis, TN 38105, USA; anna.jones@stjude.org (A.M.J.); niki.jurbergs@stjude.org (N.J.); sean.phipps@stjude.org (S.P.); elyse.heidelberg@stjude.org (R.E.H.)

**Keywords:** psychosocial care of children with cancer and their families, psychosocial concerns, universal assessment and intervention, psychoeducation, anticipatory guidance

## Abstract

**Simple Summary:**

Increased distress is a common initial response for youth and their families following the diagnosis of childhood cancer. The New Oncology Program in Psychology (NOPP) was established to provide education and information on what to expect at diagnosis, as well as ways to support coping with treatment. This project examined how NOPP participation relates to caregivers’ perceptions surrounding the navigation of difficult emotions, and of the awareness of potential cognitive/academic challenges their child may experience. Caregivers completing NOPP visits felt more informed about difficult emotions and how these may change over time. They felt more prepared for and equipped with strategies and support to manage difficult emotions. Lastly, caregivers felt more aware of and prepared for the potential effects of the diagnosis and treatment on patient cognitive/academic functioning. The results highlight important domains for universal assessment and intervention with pediatric patients and families at the time of a new cancer diagnosis.

**Abstract:**

Background/Objectives: Pediatric oncology patients and families are at risk for increased distress at diagnosis. The New Oncology Program in Psychology (NOPP) aligns psychological care with the established standards of care at diagnosis. This project aimed to evaluate NOPP and understand the differences between caregivers’ perceptions of feeling informed and prepared to navigate psychosocial concerns for those who did and did not receive psychological services at diagnosis. Methods: A survey was administered via a virtual platform. Frequency analyses summarize caregiver experiences and concerns. Mann–Whitney U tests assess the differences in caregiver knowledge and preparedness between caregivers who did and did not receive psychological services. Results: Caregivers reported difficult emotions at diagnosis and expressed concern for the impact of diagnosis and treatment across broad domains of patient functioning. Caregivers of patients who received psychology consultation felt more informed about difficult emotions and how these may change over time. They felt more prepared and equipped with strategies to manage difficult emotions. Caregivers of patients who completed a cognitive assessment also felt more informed and prepared regarding the potential effects of the diagnosis and treatment on patient cognitive/academic functioning. Conclusions: Psychological services were associated with caregivers’ positive perceptions surrounding the management of difficult emotions and with their knowledge regarding the cognitive/academic impact. The results inform the ongoing modification of NOPP.

## 1. Introduction

In 2024, it is estimated that 9620 children (birth to age 14) and 5290 adolescents (age 15–19) will be diagnosed with cancer in the United States [1]. The psychosocial impact of a cancer diagnosis on pediatric patients and their families is well-documented. The impact following a pediatric oncology diagnosis is variable and can include mood, behavior, and cognitive concerns for the patient [2,3]. Similarly, a pediatric cancer diagnosis can affect caregivers. Specifically, caregivers of pediatric oncology patients experience more symptoms of depression and anxiety following diagnosis compared to the general population [4]. While most pediatric patients and their families go on to experience healthy adjustment and coping [5], it is normative for families facing life-threatening illness to exhibit distress. There are multiple disease-related milestones throughout the cancer trajectory that are characterized by increased distress and the potential for emotional, behavioral, and social difficulties [2,4,6,7]. A targeted psychosocial assessment at these critical timepoints fosters the provision of evidence-based intervention [8], which, in turn, promotes improved adjustment and engagement in adaptive coping [9]. Diagnosis is a particularly salient timepoint for patients and families as they come to terms with a serious illness and adapt to new routines and the medical environment. As such, the timely provision of education and support is warranted to optimize coping and facilitate positive adjustment [8].

The Pediatric Psychosocial Preventative Health Model is a tiered approach to care that stipulates all families should receive universal intervention and support, and additional intervention should be provided based on need [8,10]. This model aligns with the Standards for the Psychosocial Care of Children with Cancer and Their Families (The Standards) which were published in 2015 [11] and provide a framework for the provision of psychosocial services throughout the cancer trajectory. Standards of particular importance to the current project include Psychosocial Assessment [8], Monitoring and Assessment of Neuropsychological Outcomes [12], Anticipatory Guidance and Psychoeducation [13], and Psychosocial Interventions and Therapeutic Support [9]. The Psychosocial Assessment Standard urges the assessment of all families early in the oncology experience to identify the risk and resiliency factors that may impact coping and adjustment, as well as opportunities to provide intervention to families with warranting needs [8]. Similarly, The Psychosocial Interventions and Therapeutic Support Standard stipulates that intervention and support should be available to pediatric oncology patients and their families across the cancer trajectory, including at the time of diagnosis [9]. The Monitoring and Assessment of Neuropsychological Outcomes Standard calls for cognitive surveillance for patients at risk of experiencing disease- or treatment-related changes in functioning [12]. Lastly, The Anticipatory Guidance and Psychoeducation Standard states that patients and families should be given information on all aspects of their diagnosis and treatment, including information on what they may expect throughout the pediatric cancer trajectory. This education can reduce distress and negative symptomology and improve relationships and communication between the family and medical team [13]. 

At St. Jude Children’s Research Hospital (SJCRH), the New Oncology Program in Psychology (NOPP) was developed to align psychology services with the aforementioned standards, offer standardized assessment and intervention services, and improve the psychological care of patients and their families at diagnosis. Through NOPP, all new oncology patients are offered the opportunity to be systematically and prospectively assessed and provided with appropriate intervention in response to presenting concerns near the time of diagnosis or initiation of care at SJCRH. Importantly, although the offering of these services is universal, the delivery of services is tailored and individualized to ensure that a patient’s specific disease and treatment characteristics are considered, that services are culturally responsive and demonstrate cultural humility, and that services are consistent with trauma-informed care principles. Within that context, NOPP focuses on the identification of individual and familial factors that may impact the child’s adjustment to and coping with diagnosis and treatment. Importantly, NOPP extends beyond distress screening and includes a psychology consultation for all oncology and transplant/cellular therapy patients and a brief cognitive assessment for those patients who do not receive serial cognitive monitoring as a part of their therapeutic protocol. The initial consultation through NOPP (NOPP Consult) includes a clinical interview which assesses patient/family history, current functioning, sociocultural factors, and patient health-related behaviors. Additionally, patient functioning is assessed via validated self- and parent-report measures. Universal intervention which aligns with family values and preferences, including developmentally tailored anticipatory guidance and psychoeducation, is also provided at this visit. Neurocognitive and academic functioning are assessed through NOPP via a brief, targeted evaluation (NOPP Brief Cognitive Assessment). Following the NOPP Consult and Brief Cognitive Assessment, a psychosocial support plan is developed, including plans for ongoing psychological intervention and referrals to other psychosocial disciplines, as indicated. NOPP aims to transform psychology services at SJCRH from a consult-based model to an integrated model of psychological care consistent with The Standards [14], ensuring all oncology and transplant patients receive equitable psychological support. 

NOPP was developed and evaluated following the CDC’s Framework for Program Evaluation in Public Health [15]. Stakeholder input was a cornerstone of NOPP’s early development. Prior to the launch of NOPP, the SJCRH Patient Family Advisory Council (PFAC), a group of advisers who partner with staff to share the patient and family perspective on various initiatives, provided support for NOPP and offered feedback regarding the scope of the program and timing of services. Additionally, input and feedback from patients, families, hospital administration, medical teams, psychosocial clinicians, and subject matter experts was obtained. The current project further engaged stakeholders more broadly by seeking feedback regarding NOPP from the larger patient and family community. In NOPP’s development, the senior author, psychology leadership, and hospital administration collaborated to create and support a program to systematically and prospectively provide universal assessment and intervention to all new patients within 1–2 months of their diagnosis or initiation of care. The goals of NOPP include providing an assessment and intervention to reduce distress and increase families’ perception of feeling informed, prepared, and equipped to manage potential psychosocial and cognitive sequela associated with a new pediatric oncology diagnosis. Following the implementation of NOPP, the authors deemed it critical to evaluate the impact of the program and solicit feedback from stakeholders in the context of ongoing program evaluation. Therefore, the current survey was developed with input from subject matter experts and feedback from interdisciplinary collaborators. One goal of the survey was to assess the concerns of families and identify the differences between those who did and did not participate in NOPP. The survey was distributed to stakeholders (i.e., caregivers and young adult patients) to assess their perceptions of feeling informed, prepared, and equipped to manage potential psychosocial concerns. The survey was hosted on St. Jude Voice, a virtual advisory platform where patients and families are surveyed for their input regarding clinical initiatives and other patient-care-related issues. To justify the conclusions, the survey results were statistically analyzed and interpreted. From these findings, recommendations will be made to improve the impact of the program. Additionally, this article serves to disseminate the findings and share the recommendations to the larger academic community. Lastly, the project results and the value of families’ participation will be shared with stakeholders in a newsletter to the larger St. Jude Voice community [15].

The current project represents the first effort since the launch of NOPP to understand family perceptions of psychology services near the time of diagnosis or initiation of care at SJCRH. Specifically, the current project aims to understand the psychosocial concerns and difficulties experienced by patients and their families near the time of diagnosis. The second aim of the current project is to examine the differences in feeling informed of and prepared to navigate potential psychosocial challenges between those who met with a psychologist for a consult near the time of diagnosis or initiation of care at SJCRH and those who did not. Finally, this project seeks to investigate the differences between those who completed a neurocognitive assessment near the time of diagnosis or initiation of care at SJCRH and those who did not as it relates to caregiver perceptions of feeling informed of and prepared to navigate potential cognitive/academic challenges. 

## 2. Materials and Methods

### 2.1. Human Subjects Protection

Although program evaluation projects do not require approval by the Institutional Review Board at the authors’ institution, the Institutional Review Board was consulted, and the project was determined to be non-human subject research. 

### 2.2. Materials

The survey consisted of 38 questions. A five-point Likert scale was used for most questions and collapsed during analysis into a three-point scale (strongly agree/agree, neither agree or disagree, and strongly disagree/disagree). Strongly agree and agree were indicative of endorsement of the experience.

### 2.3. Procedures

The survey was developed with input from subject matter experts and feedback from interdisciplinary collaborators to be consistent with surveys used in program evaluation projects examining clinical programs within pediatric oncology [16]. The survey was administered via an online platform (St. Jude Voice) in English. St. Jude Voice has 581 caregivers, and 41 young adult patients enrolled. The majority of enrolled caregivers identify as female (90.19%), identify as White (78.31%), reside in the United States (93.29%), and have completed treatment (53.97%) The survey link was emailed to St. Jude Voice members and remained open for three weeks. One week prior to the survey closing, a reminder was emailed to eligible caregivers and patients who had not completed the survey. No incentives were provided for participation. NOPP was gradually implemented across diagnostic oncology clinics and reached full implementation (i.e., services offered to all new oncology and transplant/cellular therapy patients) in 2023. As such, caregivers and patients on St. Jude Voice are varied regarding whether or not they were offered NOPP services. This allows for a comparison between participants who completed a Consult and/or Brief Cognitive Assessment near the time of diagnosis or initiation of care at SJCRH (i.e., within 1–2 months of arriving to SJCRH) and those who did not. Furthermore, this allowed us to understand whether those who did not participate in NOPP believe a consult and/or an assessment would have been beneficial near diagnosis. 

### 2.4. Analyses

To better understand if participation in the NOPP Consult and Brief Cognitive Assessment differed based on demographic variables (i.e., age, gender, and race), ANOVAs were utilized. For aim 1, frequency analyses were used to calculate the percentage of participants who expressed worry in the assessed domains and the percentage of individuals who expressed experiencing difficult emotions at the time of diagnosis. For aims 2 and 3, two Mann–Whitney U tests were used to compare the differences in feeling informed of and prepared to manage potential challenges for those who did and did not complete a Consult and Brief Cognitive Assessment near time of diagnosis or initiation of care at SJCRH. Those who could not recall their participation in psychology services near the time of diagnosis or initiation of care at SJCRH were not included in the analyses for aims 2 and 3. Mann–Whitney U tests were selected as the data were not normally distributed [17]. Five caregiver participants were removed prior to any analysis as they did not answer any of the questions related to the three aims. Additionally, all six young adult patients were removed, given the small sample size. 

## 3. Results

### 3.1. Demographics

In total, 115 individuals completed the survey, with 109 identified as primary caregivers for the patient and six identified as young adult patients. Given the small number of patient respondents, this report will focus only on the response of caregivers. The majority of the caregiver sample identified as White (*n* = 98) and female (*n* = 95). See Table 1 for additional demographic information. The response rates for caregivers who began the survey and completed the survey were 19.62% and 18.76%, respectively. There were no significant differences in Consult and Brief Cognitive Assessment participation based on race or gender; however, there was a significant difference in participation in the Consult and Brief Cognitive Assessment based on age (F(7,79) = 2.51, *p* = 0.022; and F(7,82) = 3.51, *p* = 0.002, respectively). Younger children were more likely to participate in the Consult and Brief Cognitive Assessment. 

### 3.2. Psychosocial Concerns and Difficulties at Diagnosis

Almost all caregivers (99.00%) reported experiencing difficult emotions at the time of their child’s diagnosis. Many caregivers expressed worry regarding the impact the diagnosis and treatment might have on the patient’s friendships and relationships (73.27%), patient’s emotional wellbeing (94.06%), patient’s behavior (80.20%), patient’s health-related behaviors (i.e., sleep, appetite, or pain; 93.07%), and patient’s cognitive skills (82.00%). The frequency of caregiver worry is depicted in Table 2. 

**Figure 1:**
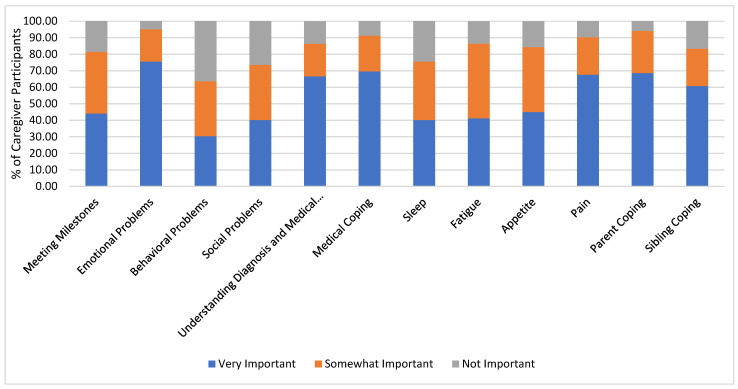
Caregiver concerns at diagnosis.

Overall, caregivers reported the following domains to be a very or somewhat important concern they held for the patient at diagnosis: meeting developmental milestones (81.37%); experiencing difficulties related to emotional (95.10%), behavioral (63.72%), or social (73.53%) functioning; understanding medical diagnosis and experience (86.27%); coping with medical experiences (91.18%); sleep (75.49%); fatigue (86.28%); appetite (84.32%); pain (90.20%); parent coping (94.12%); and sibling coping (83.33%). See Figure 1 for additional details regarding caregivers’ identified concerns at diagnosis. 

### 3.3. Psychology Consult 

Fifty-one caregivers (46.79%) reported the patient met with a psychologist for a consultation within two months of initiating care; of these caregivers, 86.00% reported the timing of this visit was appropriate. Of the caregivers who reported the patient did not meet with a psychologist or were unable to recall their participation, 80.70% (*n* = 46) thought meeting with a psychologist would have been helpful. 

Caregivers of patients who met with a psychologist, in comparison to those who did not, were more likely to report feeling informed about difficult emotions often experienced by patients and families near the time of diagnosis and how these emotional experiences may change over time (U = 490, *p* = 0.03). Relatedly, they more often reported feeling like they knew what to expect regarding their emotional experience (U = 503, *p* = 0.04) and were equipped with strategies and access to available supports to help manage difficult emotions (U = 507, *p* = 0.05). Additionally, they were more likely to report feeling informed about the potential impact of diagnosis and treatment on the patient’s emotional wellbeing (U = 520, *p* = 0.03), and equipped with strategies and access to available supports to improve the patient’s emotional wellbeing at diagnosis and throughout treatment (U = 537, *p* = 0.05). No differences were found regarding caregivers feeling informed, prepared, or equipped with strategies to manage patient concerns related to social functioning, behavior changes, and health-related behaviors (Table 3).

### 3.4. Brief Cognitive Assessment 

Almost half of caregivers (45.56%; *n* = 41) reported the patient completed a cognitive assessment within two months of diagnosis or initiating care; of these, thirty (73.17%) reported the timing of this assessment was appropriate. Of those caregivers who reported the patient did not complete a cognitive assessment or were unable to recall their participation, 28 (57.14%) thought this assessment would have been helpful. 

Caregivers who reported the patient completed a cognitive assessment, in comparison to those who did not, were more likely to report feeling informed about the potential impact of diagnosis and treatment on the patient’s cognitive skills and school performance (U = 611.50, *p* = 0.05). They were also more likely to report feeling prepared in relation to expectations regarding the patient’s cognitive skills and school performance at diagnosis and throughout treatment (U = 556, *p* = 0.01). No differences were found regarding caregivers feeling equipped with strategies and access to available supports in this domain (Table 3).

## 4. Discussion

The time of diagnosis is known to be one of increased distress for pediatric patients diagnosed with cancer and their families [2,18,19,20]. Psychologists are poised to offer a psychosocial and cognitive assessment, as well as intervention services, to mitigate distress when integrated into pediatric cancer care. NOPP offers a culturally sensitive assessment and intervention at the universal level [8,10] for all patients and their families, with goals of helping them understand what to expect throughout the cancer trajectory, learn strategies to promote patient coping and overall wellbeing, and achieve a greater understanding of access to available supports. Furthermore, NOPP facilitates the provision of psychological services at the targeted and clinical level [8,10] when indicated. 

The results of the current survey suggest that caregivers of patients who receive a psychological consultation near the time of diagnosis or initiation of care were more likely to feel informed of and prepared for challenging emotions often experienced by patients and their families. Importantly, they also noted feeling equipped with strategies and access to available supports to promote coping with these challenging emotional experiences. Similarly, caregivers of patients who received a psychology consultation were more likely to report feeling informed of and prepared for the potential impact of diagnosis and treatment on the patient’s emotional wellbeing and equipped with strategies and access to available supports to promote the patient’s emotional functioning throughout the cancer trajectory. These findings are consistent with the aims of NOPP regarding the provision of anticipatory guidance and psychoeducation related to patient and family emotional experiences and patient emotional functioning. There was no significant difference between caregivers who reported the patient received a psychology consultation and those who did not on feeling informed, prepared, and equipped across the domains of social and behavioral functioning and health-related behaviors (e.g., sleep, appetite, experience of pain, etc.), suggesting a need for improved anticipatory guidance and psychoeducation across these areas. 

Similarly, caregivers who reported the patient received a cognitive assessment near the time of diagnosis or initiation of care were more likely to express feeling informed of the potential impact of diagnosis and treatment on the patient’s cognitive functioning, as well as prepared for what to expect over time in this domain. However, there was no significant difference between those who did and did not complete a cognitive assessment on caregivers feeling equipped with strategies and access to available supports to promote the patient’s cognitive functioning and academic achievement, suggesting an important area for improved psychoeducation. 

Importantly, the target domains for NOPP assessment and intervention align with those identified as important by caregivers, supporting the use of these domains as a model for universal psychological assessment and intervention at diagnosis. 

### 4.1. Limitations

Due to limited participation within the young adult patient population, the patient perspective is not included. This is consistent with the existing literature highlighting the challenges of obtaining AYA participation in oncology research [21]. Limited AYA participation in the current project highlights an area for growth within the institutional virtual advisory platform and subsequently generated interest in intentional recruitment efforts to expand the patient voice, which are currently ongoing. While the platform remains limited to patients aged 18 and older, it is imperative that we seek and maximize direct patient feedback when developing and evaluating clinical programs. Thus, additional avenues to obtain the patient perspective for those under age 18 are needed. Furthermore, the sample was predominantly White and female. The limited racial and gender diversity limits the generalizability of the findings. Future work is needed in order to confirm the current findings in diverse samples.

While the survey assessed perceptions of psychology services near the time of diagnosis or initiation of care, defined as within 1–2 months of arriving to SJCRH, and allowed for a comparison between those who received a psychology consult and/or cognitive assessment and those who did not, it is not known whether patients who received psychological services did so as a part of NOPP or if they were coincidentally referred for psychology services within the NOPP timeframe. Regardless of the mechanism by which patients received these services, the information gleaned from the current survey will serve to inform continued improvement efforts for NOPP and psychology services offered near the time of diagnosis more broadly. 

Additionally, the current project did not assess whether families who did not complete NOPP declined to participate or were not offered NOPP based on the timeline of implementation. To ensure the services align with families’ needs and values, future modifications to NOPP should track the rate of declined participation and the reason for the decline. Further, it would be of interest to understand if those who decline differ demographically or in their perceived level of knowledge, preparedness, or feeling equipped. This information could be utilized in future quality improvement efforts—efforts that should, undoubtedly, include diverse family representation on the team.

The perception of psychological services differs based on cultural factors [22,23]. It is acknowledged that psychology services may not be perceived favorably by all families. In an attempt to reduce stigma, NOPP provides psychological services universally, to all new patients. The universal nature of NOPP is shared with patients and families when introducing the service. Nonetheless, clinicians remain mindful that sociocultural differences likely impact families within the medical and psychological setting [24,25]. Therefore, clinicians continue to engage in trainings in cultural humility and seek consultation from other mental health professionals and cultural experts within the institution. 

Universal screening and intervention aim to provide equitable and personalized resources to all families [26]. Nonetheless, providing widespread screening and intervention has not yet been implemented by all pediatric oncology centers [27]. At the majority of pediatric oncology centers, most psychological services are provided exclusively on a referral basis. Barriers to the systematic implementation of psychosocial standards of care stem from resource limitations, namely, funding, staffing, and time constraints [28]. Given the barriers, policy changes are necessary in order to ensure resources are available to provide equitable and culturally responsive care to all patients and families [28]. It is recognized that some institutions are more resource-rich than other institutions. In an effort to increase equity in access, psychoeducation and anticipatory guidance, one aspect of the NOPP universal intervention package, is widely available for all individuals regardless of treatment location [29,30,31].

### 4.2. Future Directions

The findings of the current project will be used to inform the continuous improvement of NOPP, with specific attention paid to identified areas of needed improvement, including anticipatory guidance and psychoeducation related to aspects of patient behavioral, and social, cognitive, and academic functioning, as well as health-related behaviors, including sleep, appetite, and pain. Some of these data will be used as baseline data in the Plan phase of the Model for Improvement’s Plan–Do–Study–Act framework [32]. 

While the continuous quality improvement of NOPP is underway, with results from the current project informing the Plan phase of the Model for Improvement’s first intervention cycle [32], future research is also needed in order to understand the impact of NOPP on patient outcomes, both psychological and medical, extending throughout the cancer trajectory and into survivorship.

## 5. Conclusions

The current project demonstrates that caregivers experience difficult emotions at the time of diagnosis and express concern for the impact of diagnosis and treatment across broad domains of patient functioning. These domains should be considered in the provision of universal assessment and intervention that align with the Standards for the Psychosocial Care of Children with Cancer and Their Families. The differences (and lack thereof) in feeling informed, prepared, and equipped between caregivers of patients who did and did not participate in psychology services near diagnosis will guide the continuous quality improvement of NOPP.

## Figures and Tables

**Table 1:** Demographic Information.

	*N*	%
Age at Diagnosis		
Less than 1 year old	13	12.04
1–2 years old	18	16.67
3–4 years old	8	7.41
5–7 years old	18	16.67
8–10 years old	13	12.04
11–14 years old	21	19.44
15–17 years old	13	12.04
18–23 years old	4	3.70
Missing	1	0.01
Primary Clinic		
Leukemia/Lymphoma	30	27.52
Transplant	11	10.09
Solid Tumor	33	30.28
Neuro-Oncology	32	29.36
Genetic Predisposition	3	2.75
Radiation Oncology	10	9.17
Survivorship	11	10.09
Ethnicity		
White	98	89.91
Black	3	2.75
Hispanic	4	3.67
Asian/Pacific Islander	3	2.75
Native American	0	0.00
Prefer not to answer	1	0.01
Gender		0.92
Female	95	87.16
Male	13	11.93
Other	1	0.92

Note. Participants were permitted to belong to multiple primary clinics. Therefore, the percentage of individuals in primary clinic sums to greater than 100%.

**Table 2:** Caregiver Worry for Impact of Diagnosis and Treatment on Patient Functioning.

	*n*	%
Friendships and relationships	74	73.27
Emotional wellbeing	95	94.06
Behavior	81	80.20
Health-related behaviors (sleep, appetite, or pain)	94	93.07
Cognitive skills	82	82.00

**Table 3:** Caregiver Perceptions of Being Informed, Prepared, and Equipped at Diagnosis. * *p* ≤ 0.05.

	Consult		No Consult		Brief Cognitive Assessment		No Brief Cognitive Assessment				
	M	SD	M	SD	M	SD	M	SD	*U*	*p*	η^2^
Informed											
Difficult emotions and the emotional experience might change	1.67	0.77	2.11	0.83					490.00	0.026 *	0.06
Impact on the friendships and relationships	2.00	0.84	2.36	0.83					526.00	0.070	0.04
Emotional wellbeing	1.58	0.77	2.03	0.89					520.00	0.025 *	0.06
Impact on the patient’s behavior	1.94	0.80	1.97	0.87					699.50	0.904	0.00
Impact on the patient’s sleep, appetite, and/or experience of pain	1.47	0.73	1.63	0.81					601.50	0.356	0.01
Impact on the patient’s thinking skills and school performance					1.54	0.79	1.90	0.86	611.50	0.047 *	0.05
Prepared											
Regarding the emotional experience adjusting to diagnosis and treatment	1.94	0.85	2.6	0.83					503.00	0.038 *	0.06
Regarding friendships and relationships with others	1.98	0.78	2.25	0.80					556.00	0.143	0.03
Regarding the emotional wellbeing	1.85	0.82	2.17	0.83					573.00	0.109	0.03
Regarding the patient’s behavior	2.02	0.83	2.27	0.78					615.00	0.197	0.02
Regarding the patient’s sleep, appetite, and/or experience of pain	1.62	0.83	1.93	0.91					548.50	0.131	0.03
Regarding the patient’s thinking skills and school performance					1.56	0.79	2.07	0.91	556.00	0.011 *	0.08
Equipped with Strategies and Supports											
Manage difficult emotions	1.94	0.80	2.32	0.77					507.50	0.045 *	0.05
Improve friendships and relationships with others	2.04	0.76	2.29	0.85					563.00	0.165	0.03
Improve emotional wellbeing	1.77	.78	2.17	0.87					537.00	0.046	0.05
Increase positive behavior	1.83	0.78	2.17	0.87					563.50	0.088	0.04
Improve sleep, appetite, and/or pain	1.56	0.81	1.83	0.83					545.00	0.117	0.03
To help with thinking skills and school performance					1.64	0.81	1.93	0.88	657.00	0.137	0.03

## Data Availability

Data available upon request.
